# Supplementary material for: Contribution of apical and basal dendrites to orientation encoding in mouse V1 L2/3 pyramidal neurons
Source: Nat Commun. 2019 Nov 26;10:5372. doi: 10.1038/s41467-019-13029-0 (PMC6879601; doi:10.1038/s41467-019-13029-0)
Supplement: Supplementary file 1 — Supplementary Information [file 41467_2019_13029_MOESM1_ESM.pdf]

Contribution of Apical and Basal Dendrites to Orientation Encoding in Mouse V1 L2/3 Pyramidal Neurons

Park et al., Nature Communications, 2019

Supplementary Figure 1. Visual confirmation of apical dendrite ablation.

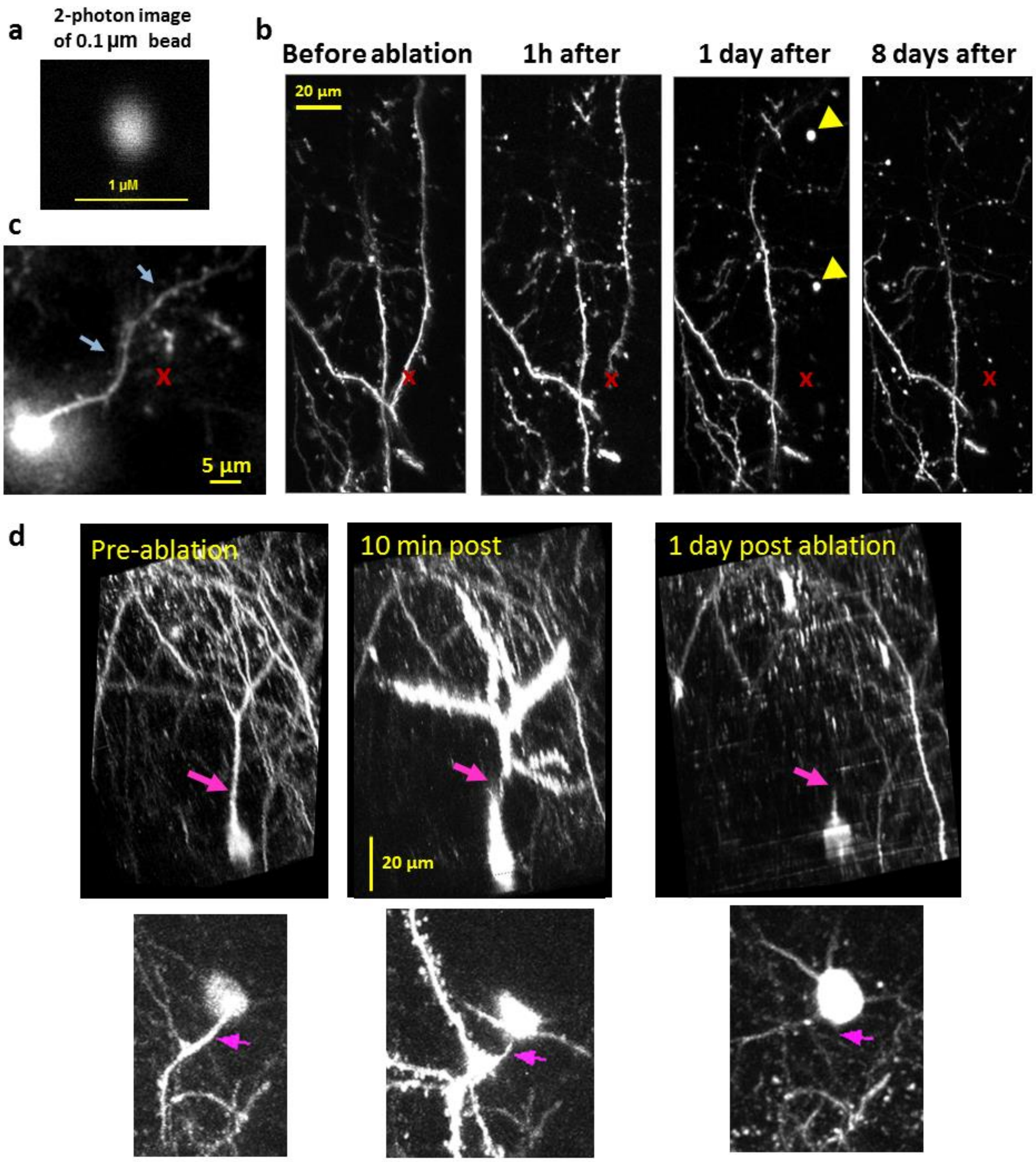

(a) 2-photon image of a 0.1 micron fluorescent bead, revealing the resolving power (and point scan size) of the microscope to be about 0.4 μm in the X-Y plane (~1.2 μm in Z). **(b)** 5-μm-thick maximum Z-projection of apical dendrites of a GFP expressing L2 pyramidal neuron imaged in vivo from a thy1-GFP mouse before and 1 hour, 1 day and 8 days after ablation. A point scan with 800nm wavelength, 200ms duration, and ~150 mW power was applied to the point marked with the red 'x'. Dendritic segments distal to ablation point developed a beads-on-a-string appearance within 1 hour post-ablation, and disappeared by 24 hours. GFP-filled remnants of the ablated dendrite were visible 24 hours after ablation (yellow arrows), but disappeared by 8 days. Dendrites near the

ablation point, even adjacent branches from the same neuron, showed no change in morphology post-ablation. **(c)** Single slice through an example apical ablation site ('red x'), acquired 5-days post-ablation (target neuron tuning curve shown in Figure 2a-c). GCaMP-labeled dendrites from nearby control neurons did not show any fine-scale changes following ablation, even ~5 μm away from the ablation site (pale blue arrows). **(d)** Side-projection (top) and top-projection (bottom) of an example neuron before (left), 10 minutes after (middle) and 1 day after 2-photon ablation of the apical dendrite. Pink arrow indicates ablation point. Neuron increased its fluorescence levels immediately after ablation likely due to electrolyte influx through the instantaneous injury caused by ablation. By 1 day following ablation target neurons' fluorescence has returned to baseline, and only a short remnant of the apical dendrite remains.

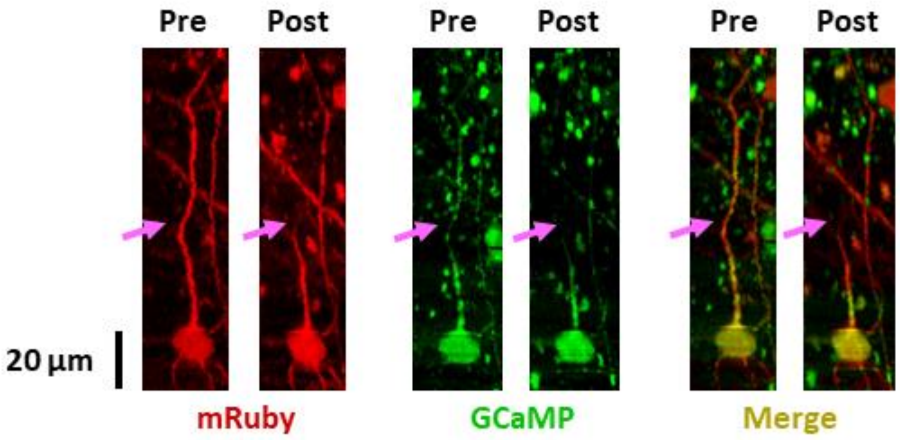

Supplementary Figure 2. Confirmation of ablation by co-expression of red fluorophore mRuby with GCaMP. Custom engineered virus (AAV8-flex-mRuby-2A-GCaMP6s) was co-injected with low titer (1:80,000) AAV1-CaMK2-Cre to enable co-expression of an activity-independent red fluorophore with GCaMP6. Side-projection of target ablated neuron in the red channel (left), green channel (middle) and merged channels (right), showing overlap of GCaMP6 and mRuby expression in the neuron before and 5 days after ablation of the apical dendritic tuft (pink arrow depicts ablation point). Vertical variation in the green signal (middle panels) is due to fluctuations in GCaMP fluorescence which reflect activity of the cell during z stack acquisition.

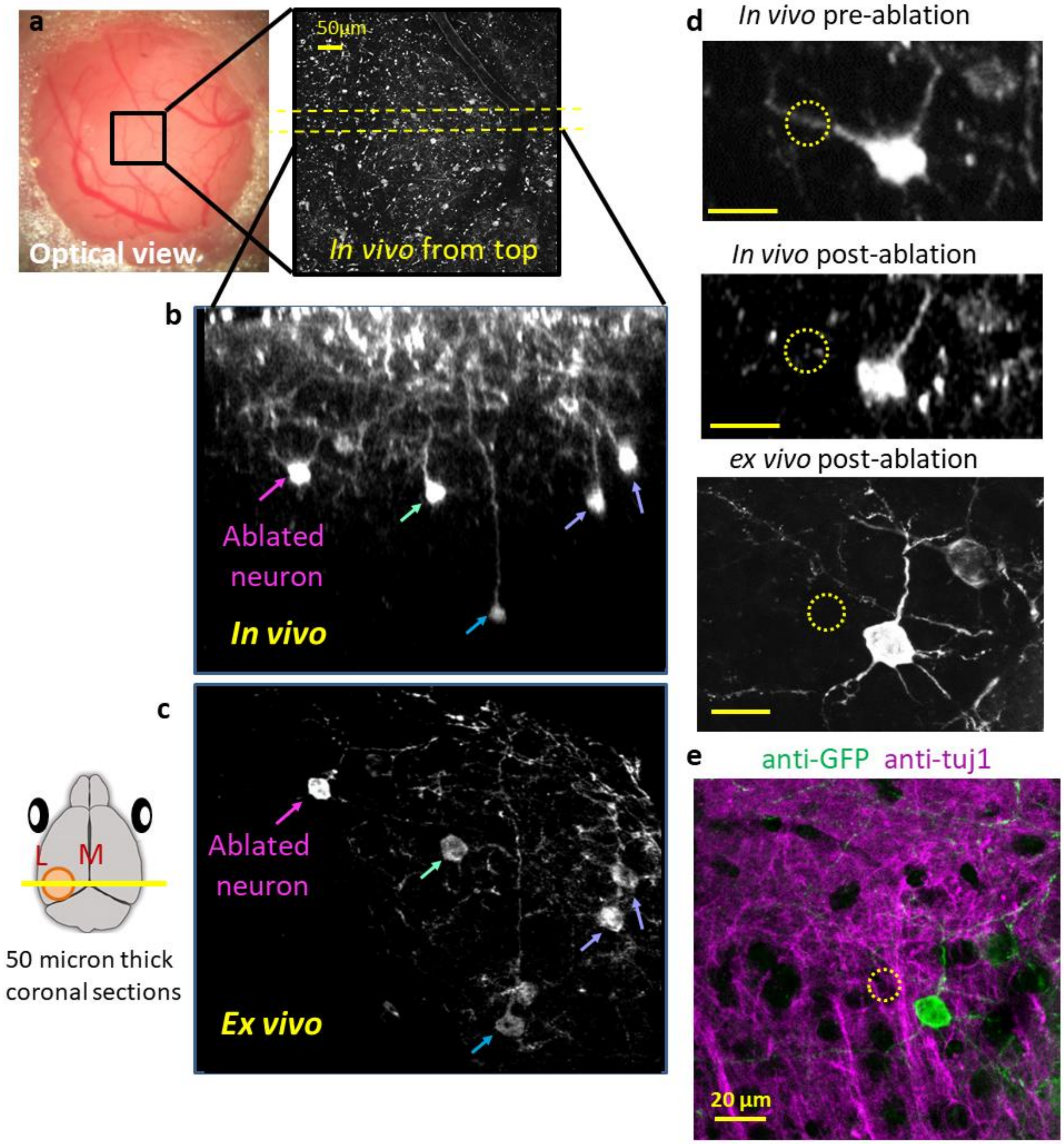

Supplementary Figure 3. Post-hoc immunohistochemical confirmation of ablation and assessment of damage at ablation site.

Dendrites of five neurons in this brain were successfully ablated. Cells were chosen at the edge of the ~400-micron-diameter sphere of GCaMP expression to ease locating them post-hoc. One day after ablation, the brain was fixed in paraformaldehyde, and 50-micron-thick coronal sections were made through the visual cortex, tracking each slice from posterior to anterior. Slices were immunostained with anti-GFP and anti-Tuj1 antibodies to visualize GCaMP-labeled and unlabeled neuronal processes, respectively. **(a)** _Left:_ Optical image of craniotomy and imaging window. _Right:_ two-photon z projection of the boxed region from left panel. Note the same surface blood vessels are visible in each image. **(b)** *In vivo* coronal side view of an ablated neuron (pink arrow) and identified neighboring labeled neurons (other arrows). **(c)** *Ex vivo* image of same neurons visualized in vivo in panel b. **(d)** Zoomed-in views of the ablated neuron

before ablation in vivo (top) after ablation in vivo (middle) and ex vivo (bottom). Note the similar orientations of the remaining dendrites emanating from the soma, as well as the location of a neighboring neuron. Yellow circle centered at ablation site. Diameter of the yellow circle is 12 micron in (d) and (e). All scale bars in (d) and (e) are 20 microns. Images acquired in vivo (top two panels) were 3D rendered and rotated to optimally display the ablated dendrite. Apparent differences in structure sizes are due to differences in viewing angle. **(e)** Immunohistochemical assessment of damage at ablation site. Green shows anti-GFP signal (amplifying GCaMP), magenta shows anti-Tuj1 signal (antibody specific to axonal and dendritic microtubules). No clear sign of disturbed processes were observed around the ablation site as confirmed previously with EM (See Fig. 2 of [1]). Note that the black regions that appear to be within the dotted circle (ablation target) do not actually represent lesions but unstained regions corresponding to cell bodies appreciated better on a different plane. These are more clearly represented in animated Z-stacks, Supplementary Movie 3.

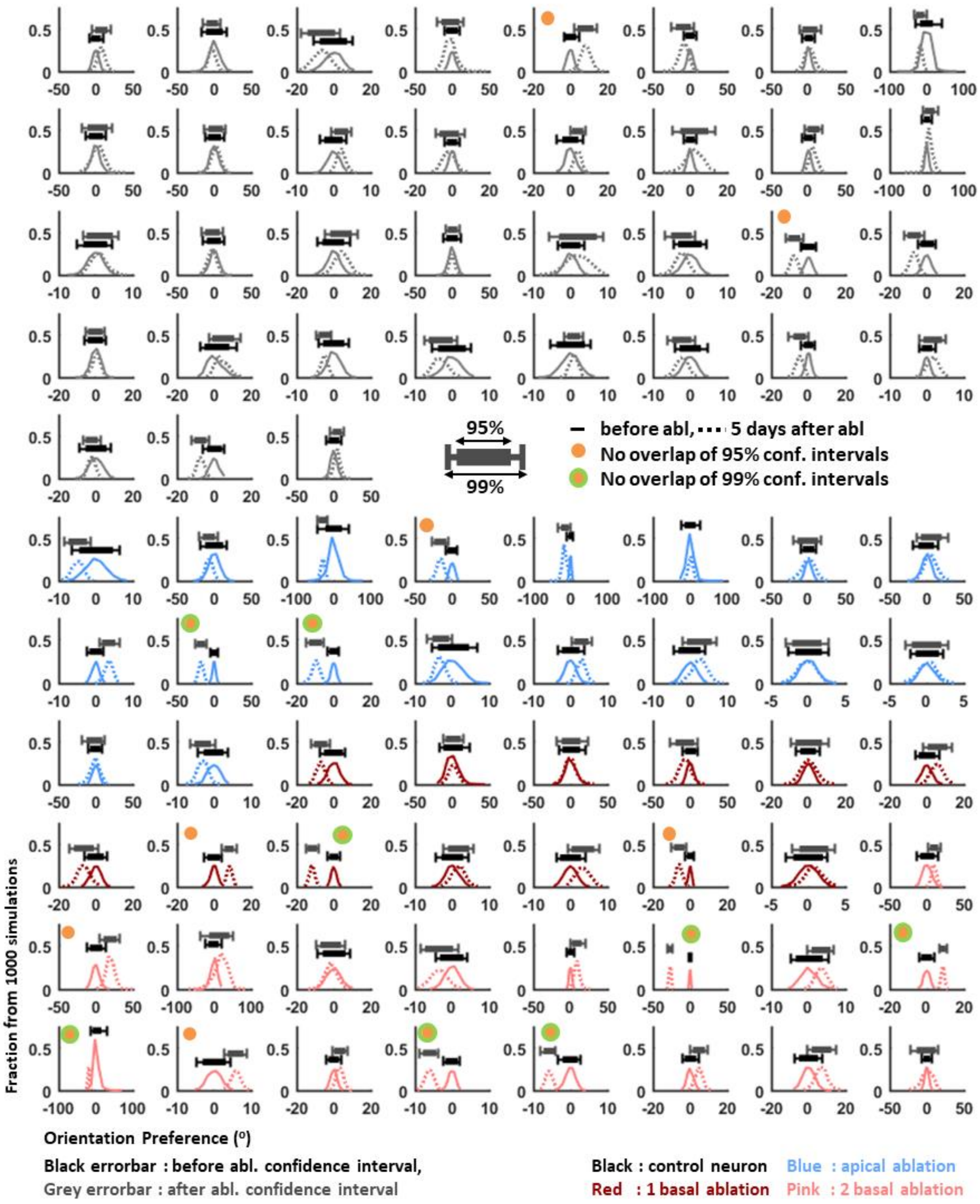

Supplementary Figure 4. Distribution of bootstrap estimates of neurons' orientation preferences before and after ablation. Histogram of the distribution of bootstrapped estimates of the orientation preferences before (solid line) and after ablation (dotted line) for neurons with apical dendrite (blue, n=18), 1 basal dendrite (dark red, n=13), 2 basal dendrite (pink, n=17) ablation and control neurons (black, n=35) from 1000 simulations. Five single-trial responses were randomly sub-selected from the 20-50 trials acquired per orientation to generate simulated tuning curves. The preferred orientation was calculated from the von Mises-fit orientation tuning curve based on the sub-sampled data. This was

repeated 1000 times to generate confidence intervals for the estimate of the preferred orientation pre and post ablation per neuron. Note that all neurons have a narrow distribution for the orientation preference even after ablation, which indicates highly robust tuning even after dendrite ablation. Distributions of the orientation estimates were shifted to have the mean of the preferred orientation estimates before ablation at 0°. Errorbars above the histograms indicate the 95% (filled box) and 99% (tick to tick) confidence intervals before (black) and after (grey) ablation. A yellow dot indicates that there is no overlap between the 95% confidence intervals of the pre-and post- ablation orientation preference estimates. Green ring indicates that there is no overlap between 99% confidence intervals of the pre- and post-ablation orientation preference estimates. 7 out of 17 two-basal-dendrite-ablated neurons showed significant separation of the distribution of orientation preference estimates before and after ablation (yellow dot) while 3 out of 13 one-basal-dendrite-ablated, 3 out of 18 apical-dendrite-ablated, and 2 out of 35 control neurons did. The frequency of detecting neurons with significant difference in preferred orientation pre- and post- ablation was significantly different than controls as assessed by the χ^2 statistic. Specifically, for 2 basal vs control is χ^2=10.1 (p=0.0015), while p was not significant for either 1-basal (p=0.08) or apical dendrite ablation (p=0.19) versus controls.

Supplementary Table 1. Chi-square test comparing control and ablated neurons for finding neurons with significant shift in preferred orientation.

Chi-square test comparing the chance of having neurons with significant shift in orientation preference	Apical vs Control p-value (χ^2)	1Basal vs Control p-value (χ^2)	2Basal vs Control p-value (χ^2)
95% confidence interval	0.19 (1.67)	0.08 (3.1)	0.0015 (10.1)

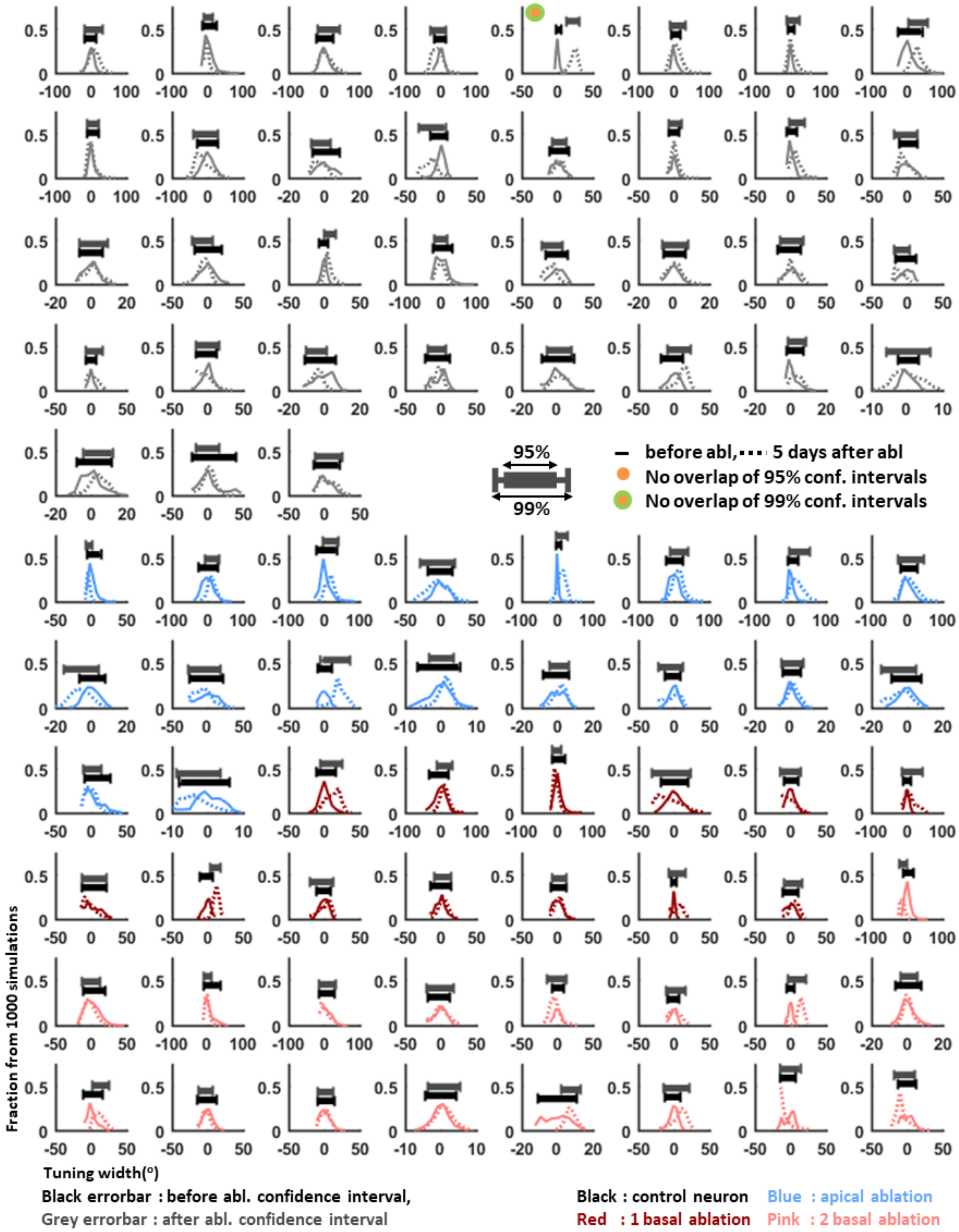

Supplementary Figure 5. Distribution of bootstrap estimates of neurons' tuning width before and after ablation. Histogram of the distribution of bootstrapped estimates of tuning width before (solid line) and after ablation (dotted line) for neurons with apical dendrite (blue, n=18), 1 basal dendrite (dark red, n=13), 2 basal dendrite (pink, n=17) ablation and control neurons (black, n=35) from 1000 simulations. Five single-trial responses were randomly sub-selected from the 20-50 trials per orientation to generate

simulated tuning curves. The tuning width was calculated from mean orientation tuning curve based on the sub-sampled data. This was repeated 1000 times to generate bootstrap confidence intervals for the estimate of the tuning width pre- and post -ablation per neuron. Errorbars above the histograms indicate the 95% (filled box) and 99% (tick to tick) confidence intervals before (black) and after (grey) ablation. A yellow dot indicates that there is no overlap between the 95% confidence intervals of the pre-and post-ablation tuning width estimates. Green ring indicates that there is no overlap between 99% confidence intervals of the pre- and post-ablation tuning width estimates. Only 1 out of 83 neurons (in the control condition) showed a significant shift in tuning width by this measure.

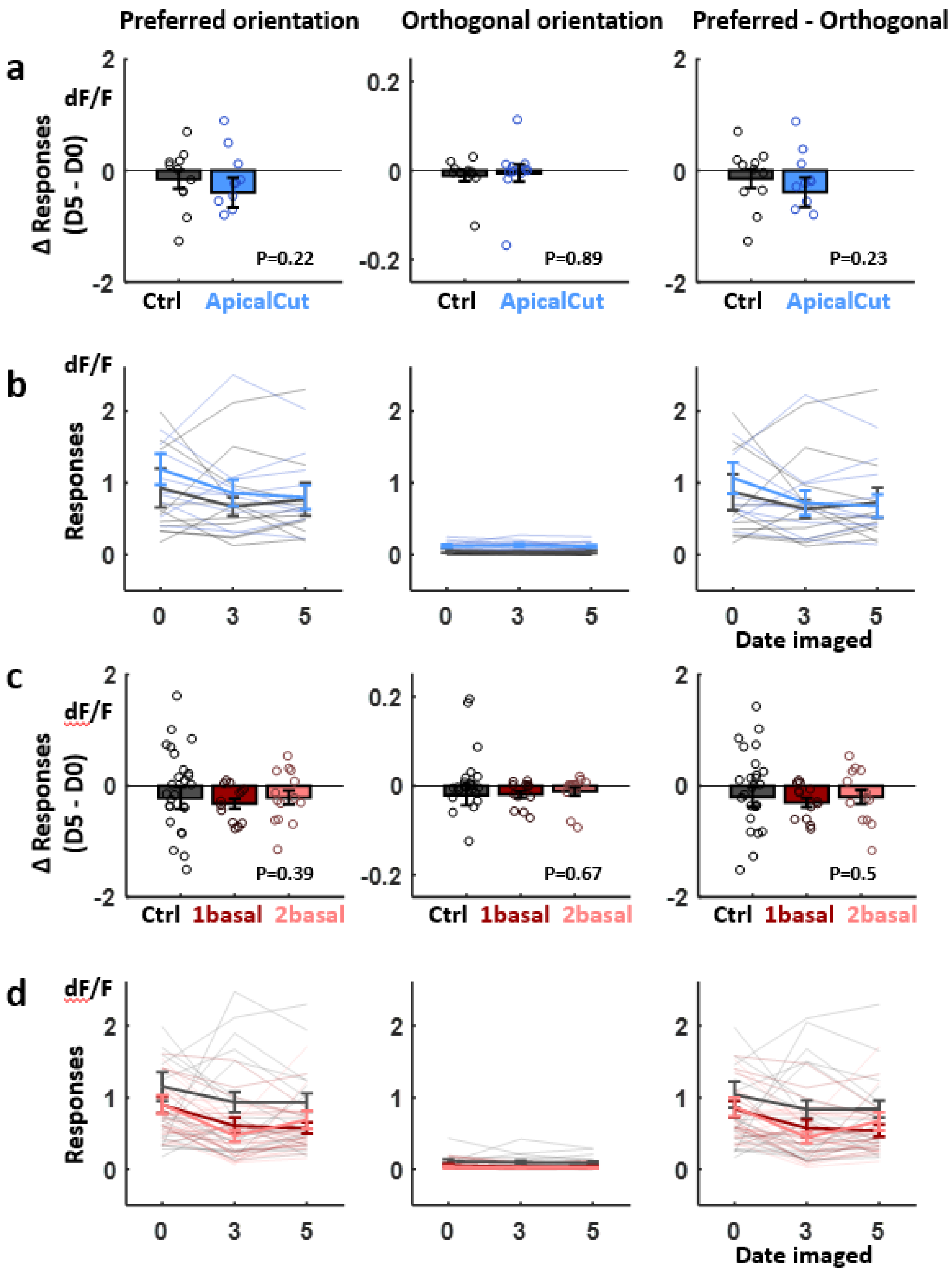

Supplementary Figure 6. Response amplitude across days in ablated neurons are comparable to those of control neurons. a, mean±SEM of changes in responses to preferred orientation (left), orthogonal orientation (center) and gain [(response to preferred orientation) – (response to orthogonal orientation), right] before and 5 days after apical dendrite ablation. P-values are from Kruskal-Wallis test. Outputs are

$\chi2(1,33)=1.49$, $p=0.22$ (preferred orientation, left), $\chi2(1,33)=0.02$, $p=0.89$ (orthogonal orientation, center) and $\chi2(1,33)=1.41$, $p=0.23$ (pref-ortho). **b,** individual lines represent mean response of a single neuron from 20-30 trials in response to its preferred (left), orthogonal orientation (center), or the differences between preferred and orthogonal orientations (right). Day 0 corresponds to pre-ablation. Thick lines are averaged value across mean responses of individual neurons. Error bar is SEM across neurons. (a)-(b), blue : apical dendrite ablation, black/grey: control neurons imaged together with ablated neurons. **c,** mean±SEM of changes in responses to preferred orientation (left), orthogonal orientation (center) and gain [(response to preferred orientation) – (response to orthogonal orientation), right] before and 5 days after 1 basal and 2 basal dendrite ablation. P-values are from Kruskal-Wallis with Tukey test for multiple comparison. Outputs are $\chi2(2,62)=1.9$, $p=0.39$ (preferred orientation, left), $\chi2(2,62)=0.8$, $p=0.67$ (orthogonal orientation, center) and $\chi2(2,62)=1.4$, $p=0.49$ (pref-ortho). **d,** figures for basal dendrite ablation in the same format with (b). (c)-(d), dark red: 1 basal dendrite ablation, pink: 2 basal dendrite ablation, black/grey: control neurons.

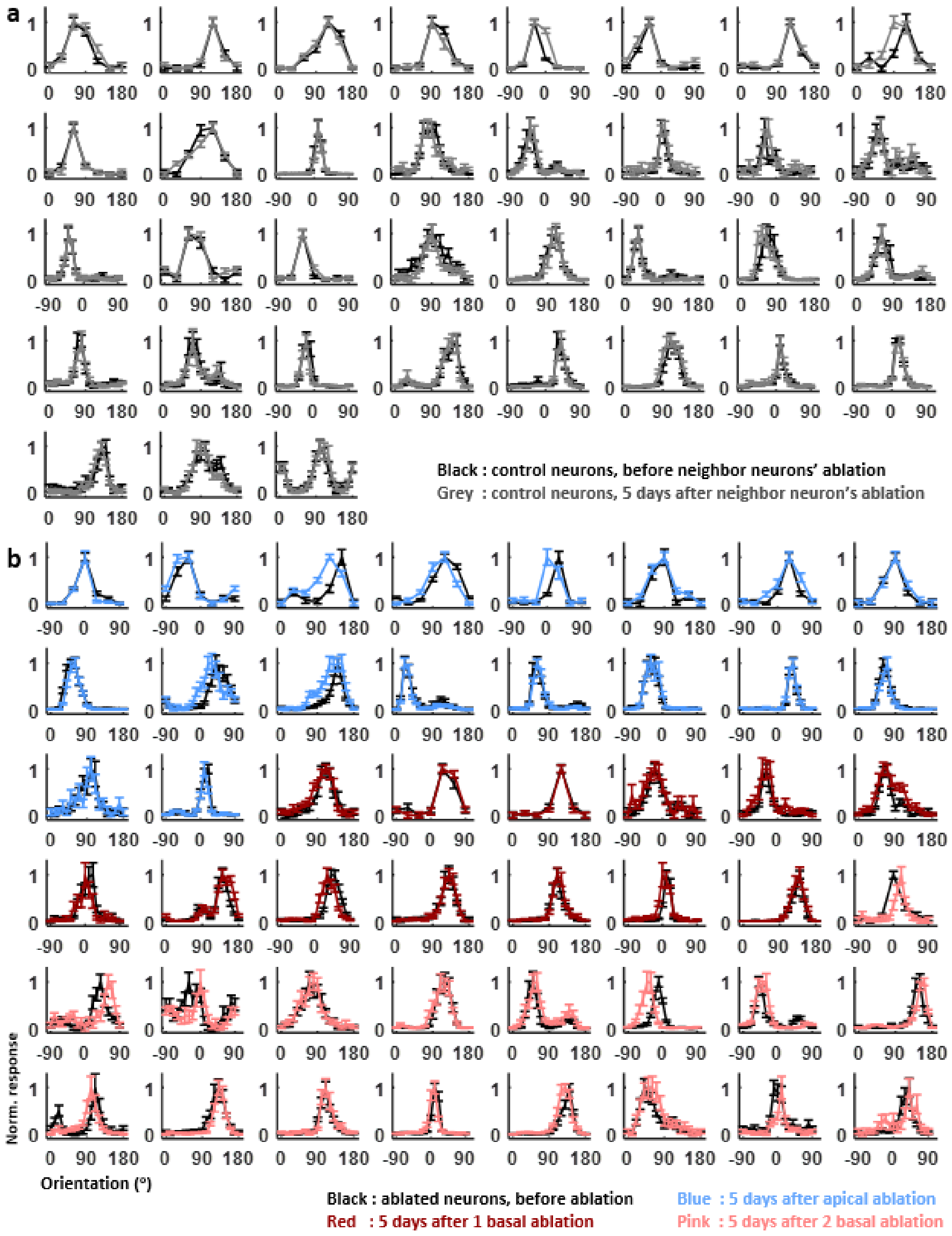

Supplementary Figure 7 Peak normalized orientation-tuning curves of control and ablated neurons. All tuning curves in (a) and (b) were baseline-subtracted and peak-normalized for illustration purposes only. **(a)** Tuning curves of control neurons before

(black) and 5 days after (grey) neighbor neurons' ablation. **(b)** Tuning curves of ablated neurons. Pre-ablation tuning curves are in black. 5 days post-ablation tuning curves are in blue (apical dendrite ablation), red (single primary basal dendrite ablation) or pink (double primary basal dendrite ablation). Although there are occasional L2/3 pyramidal neurons that show small shifts in orientation preference following apical dendrite ablation (3/18), control neurons (2/35) or single basal dendrite ablation (3/13), only neurons with 2 basal dendrite ablations show significant orientation preference shift on average (7/17, ~12.5° on average, see Fig. 3f, see Supplementary Fig. 4 for neurons with significant shift in preferred orientation upon ablation). Furthermore there was no significant change in tuning width or orientation selectivity index on average across each condition (Fig. 3g,h).

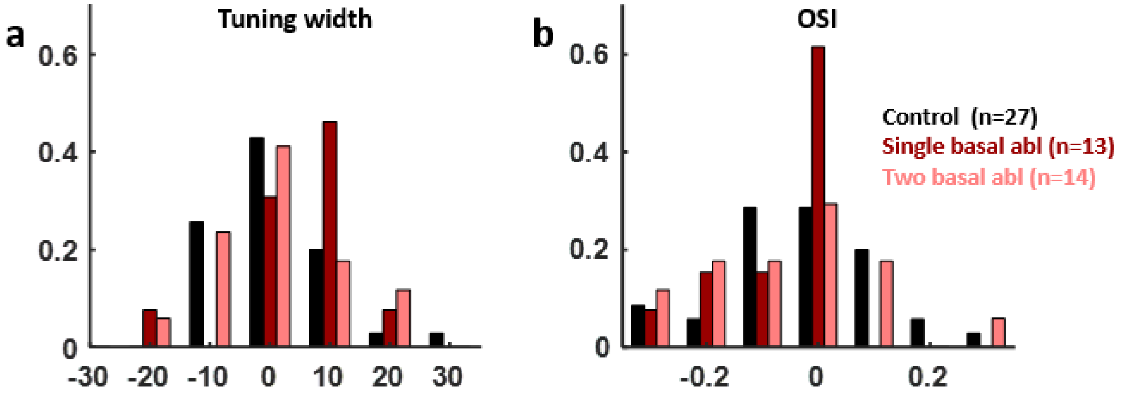

Supplementary Figure 8. Histogram of the change in tuning width (a) **and orientation selectivity index** (b) for control (black, n=35), one (dark red, n=13), and two (pink, n=17) primary basal dendrites ablated neurons. (a) Tuning width: $\chi^2(2,62)=1.44$, p=0.49, (b) OSI: $\chi^2(2,62)=0.59$, p=0.75, Kruskal-Wallis test.

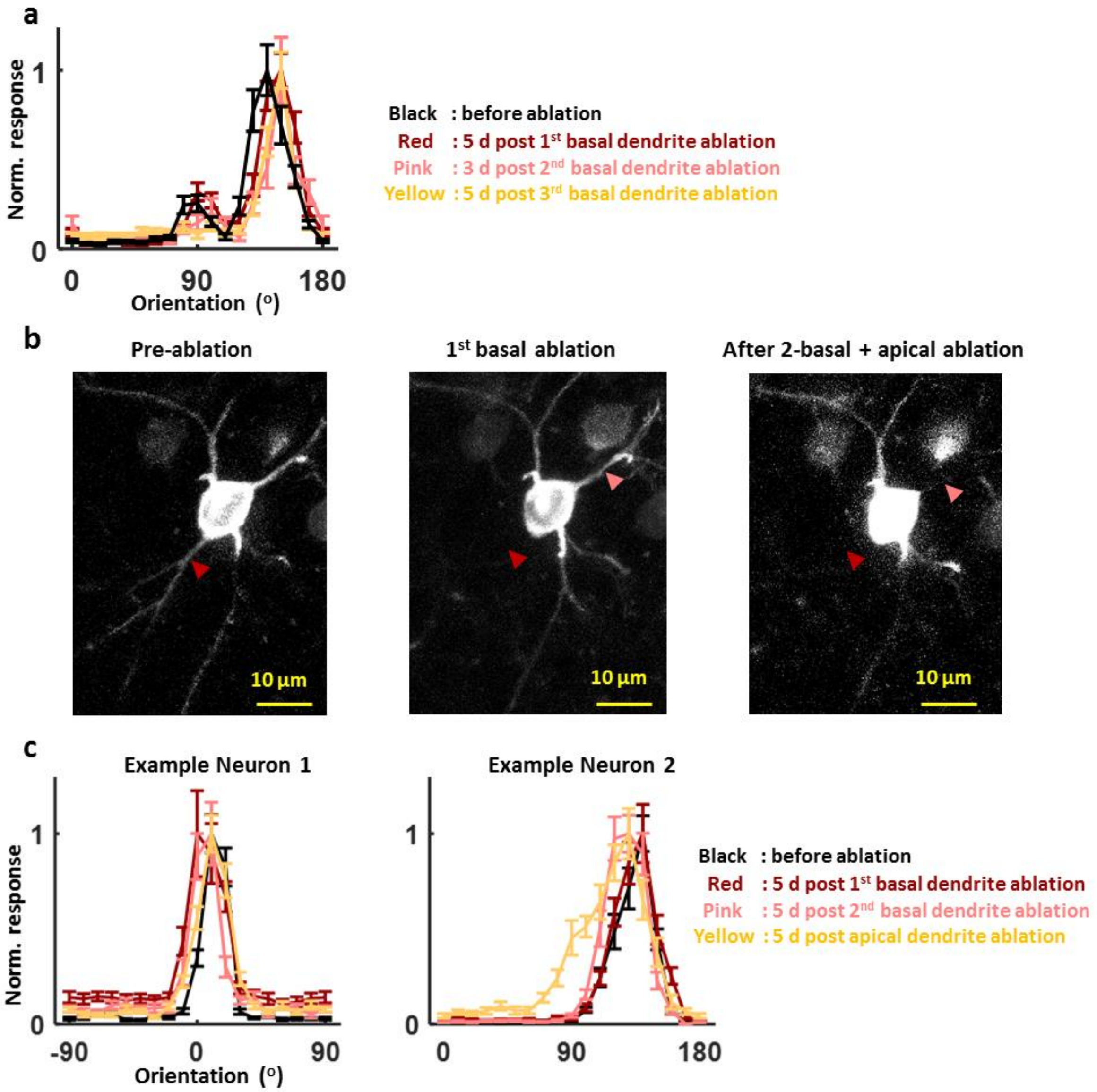

Supplementary Figure 9. Stable orientation preference following multiple dendrite ablation. (a) Peak-normalized tuning curves of the neuron with three sequential basal dendrite ablation. The first ablation had little effect. The second ablation caused the cell's tuning curve to shift by ~20 degrees. After the third ablation the tuning curve shift of the main peak remained unchanged. **(b)** Z projections depicting soma and basal dendrites showing structure of example neuron before ablation (left) after 1 basal ablation (middle) and after 2-basal+apical ablation (right). Red arrow points to the basal dendrite that was ablated first. Pink arrow points to the basal dendrite that was ablated second. Apical dendrite ablation was assessed in other z-stack images (Supplementary Movie 4) and apical dendrite is not depicted in these images. **(c)** peak-normalized tuning curves of the neuron after two basal dendrite followed by apical dendrite ablation.

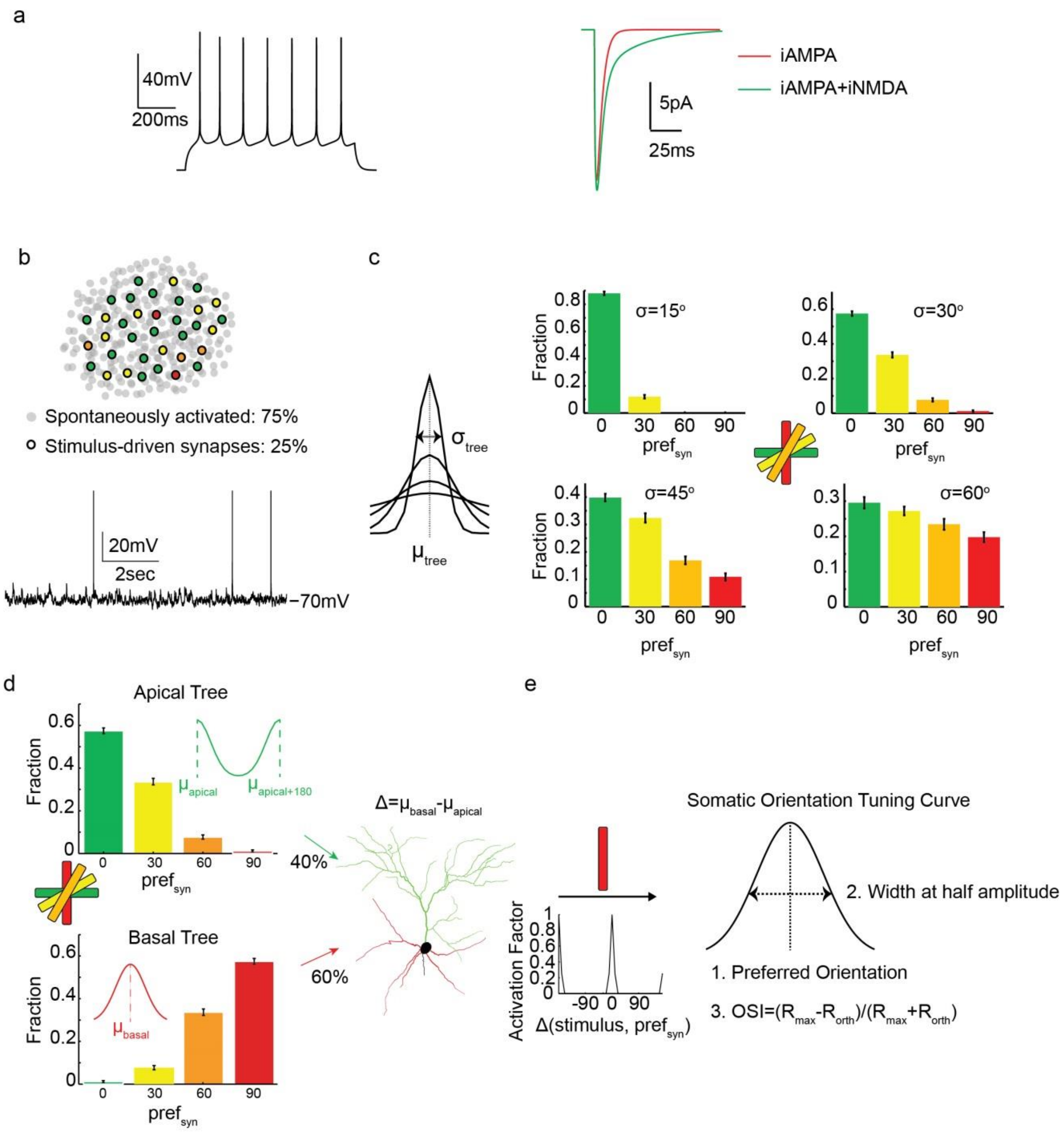

Supplementary Figure 10. Single neuron model of orientation preference.

(a) *Left:* Model response to 900ms current clamp (0.16nA). Compare with experimental of Rhie et al.[2]. ***Right***: Modelling properties of mEPSCs under control conditions (combined AMPA and NMDA current, green trace) and in the presence of APV (modelled as iAMPA, red trace). Compare with experimental data of Myme et al.[11]
(b) *Top:* From the synaptic pool, 25% were stimulus driven (colored dots) and the rest were activated from background noise[3]. *Bottom:* Indicative trace showing fluctuations of the membrane potential in the presence of background synaptic activity. The membrane potential rests above -70mV. Spikes are truncated for visualization purposes.
(c) Each tree was characterized by a $\mu_{tree} \pm \sigma_{tree}$ that determined the individual orientation preferences of activated synapses. Right: Fraction of synapses with a specific orientation preference ($pref_{syn}$), for σ_{tree}=15°, 30°, 45° and 60°. Bin width corresponding to the value reported on the x-axis is ±10°. **(d)** Exemplar case of the simulation setup, for $\Delta(\mu_{basal},\mu_{apical})$ =90°, showing the distributions

of $pref_{syn}$ of synapses for the apical (top) and basal (bottom) trees. Note that in this example, the dendritic trees have the same σ, but different μ. Synapses of each tree are assigned with a preferred orientation and then uniformly distributed along the apical and basal dendrites. **(e)** *Left:* When a stimulus is ‘presented’, the activation pattern of each synapse depends on the difference of $pref_{syn}$ and the presented stimulus (activation factor). *Right:* Metrics of the resulting tuning curve at the soma.

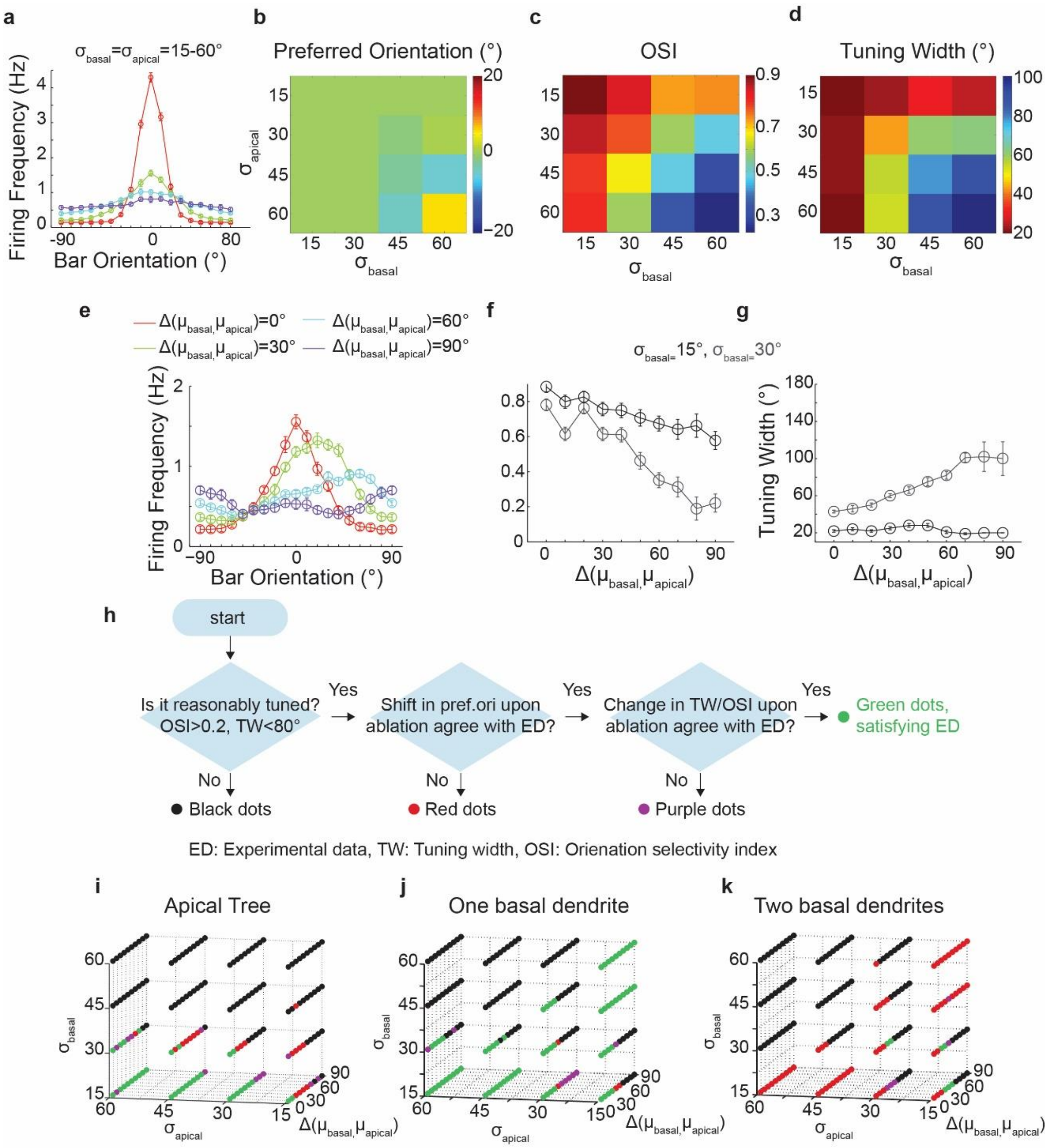

Supplementary Figure 11. Input structures that replicate the experimental results. **(a)** Average orientation-tuning curves of 10 neurons for different σ. $\Delta(\mu_{basal},\mu_{apical})$ =0° across four conditions. σ_{apical}=σ_{basal}=15° (red), σ_{apical}=σ_{basal}=30° (green), 45° (blue) or 60 (purple). **(b)** Heat map plots of preferred orientation (left), OSI (center) and tuning-width (right) of the model neuron for different σ_{basal} and σ_{apical}. $\Delta(\mu_{basal},\mu_{apical})$ was fixed at 0° and μ_{trees} was arbitrarily set at 0°. **(c)** Average orientation-tuning curves of 10 neurons with varying $\Delta(\mu_{basal},\mu_{apical})$**,** ($\sigma_{apical}$=$\sigma_{basal}$=30°). **d-e,** OSI **(d)** and tuning-width **(e)** as a function of Δ at σ_{basal}=15° (black) and σ_{basal}=30° (grey). For both σ_{apical}=30°. Error bars are SEM. Note that plots in a-e are from model neurons that are intact, not ablated. **(f)** Heat map depiction of pre- and post- apical dendrite ablation difference in preferred orientation (left), OSI (center) and tuning-width (right) when σ_{apical}=30°, for σ_{basal}={15°, 30°, 45°, 60°} and $\Delta(\mu_{basal},\mu_{apical})$={0°,…,90°}. **(g)** Logic diagram of categorizing

simulation results for 3D heat map figure h-j. *Black dots:* parameter space that results in tuning curves with OSI<=0.2 and tuning-width >=80° before or after ablation in more than 30% of the simulated neurons. If differences in preferred orientation before and after ablation agree with experiments (i.e. <10° mode shift for apical and single basal cuts and ≥10° mode shift for two basal cuts), they are further categorized as green or purple dots based on their post ablation tuning-width and OSI. Thresholds to classify a change in OSI or tuning-width correspond to the mean+1std of the experimental data and are mean OSI change>0.2 and mean tuning-width change>10°. Model neurons that did not agree with experimental shifts in orientation preference are shown in red. **h-j** Summary of dendrite ablation model results for apical (h), single basal (i) and double basal (j) dendrite ablation. See (g) for logic of categorizing simulation results. For each simulated neuron (n=10), every combination of one or two basal dendrites was removed. **(i)** and **(j)** *Green dots:* mode shift in preferred orientation is <10° without significantly altering tuning-width and OSI. *Red dots:* mode shift in preferred orientation is ≥10°. *Purple dots*: no mode shift change in preferred orientation WITH changes in tuning-width or OSI (see (g)). **(j)** *Green dots:* mode shift in preferred orientation is ≥ 10° AND tuning-width /OSI remain unchanged upon two basal dendrite cut. Max mode shift in preferred orientation among green dot conditions is 10°. *Red dots:* no shift in the preferred orientation (<10°) upon two basal cut. *Purple dots*: mode shift in the preferred orientation (≥10°) WITH changes in tuning-width or OSI (see legends for (g) for detail). In all h-j, only the green dots satisfy the experimental data. Note that there is no parameter space combination that simultaneously satisfies the experimental data of apical tree, one basal and two basal dendrites ablation. Therefore, this necessitates the introduction of differentially tuned basal dendrites. See Figure 5 and manuscript.

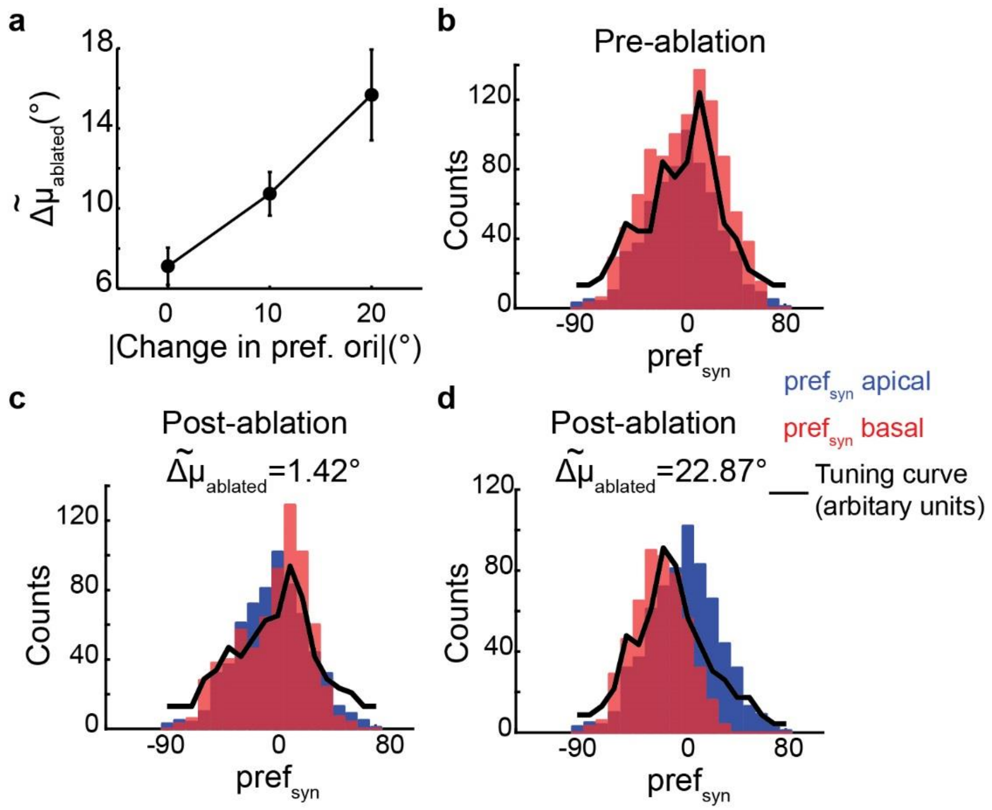

Supplementary Figure 12. Mean orientation preference change in basal dendrites after two basal dendrite ablation correlates with preferred orientation change at the soma (in the model). (a) The change in length-normalized mean orientation preference of the input across basal dendrites following two basal dendrite ablation ($\widetilde{\Delta\mu}_{\mathrm{ablated}}$; see Supplementary Methods) as a function of the change in preferred orientation of the model neurons. Note that, as expected, they are highly correlated. Simulation parameters: Δ_μ=40°, σ_{apical}=30°, σ_{basal}= 15°. Error bars: SEM. **(b)** Histogram of single synaptic preferred orientation ($pref_{syn}$) of basal (orange) and apical dendritic trees (blue) of an exemplar neuron. Overlap appears as dark red. *Black Curve:* Resulting tuning curve of this neuron (arbitrary units). **(c)** For the same simulated neuron, removing two dendrites, resulting in small $\widetilde{\Delta\mu}_{\mathrm{ablated}}$ (1.42°) does not alter orientation-tuning. **(d)** However, removing two dendrites resulting in large $\widetilde{\Delta\mu}_{\mathrm{ablated}}$ (22.87°) alters both input structure and orientation-tuning. $\widetilde{\Delta\mu}_{\mathrm{ablated}}$ significantly increased with increasing post-ablation shift in orientation preference (p=0.0087, Kruskal-Wallis test)

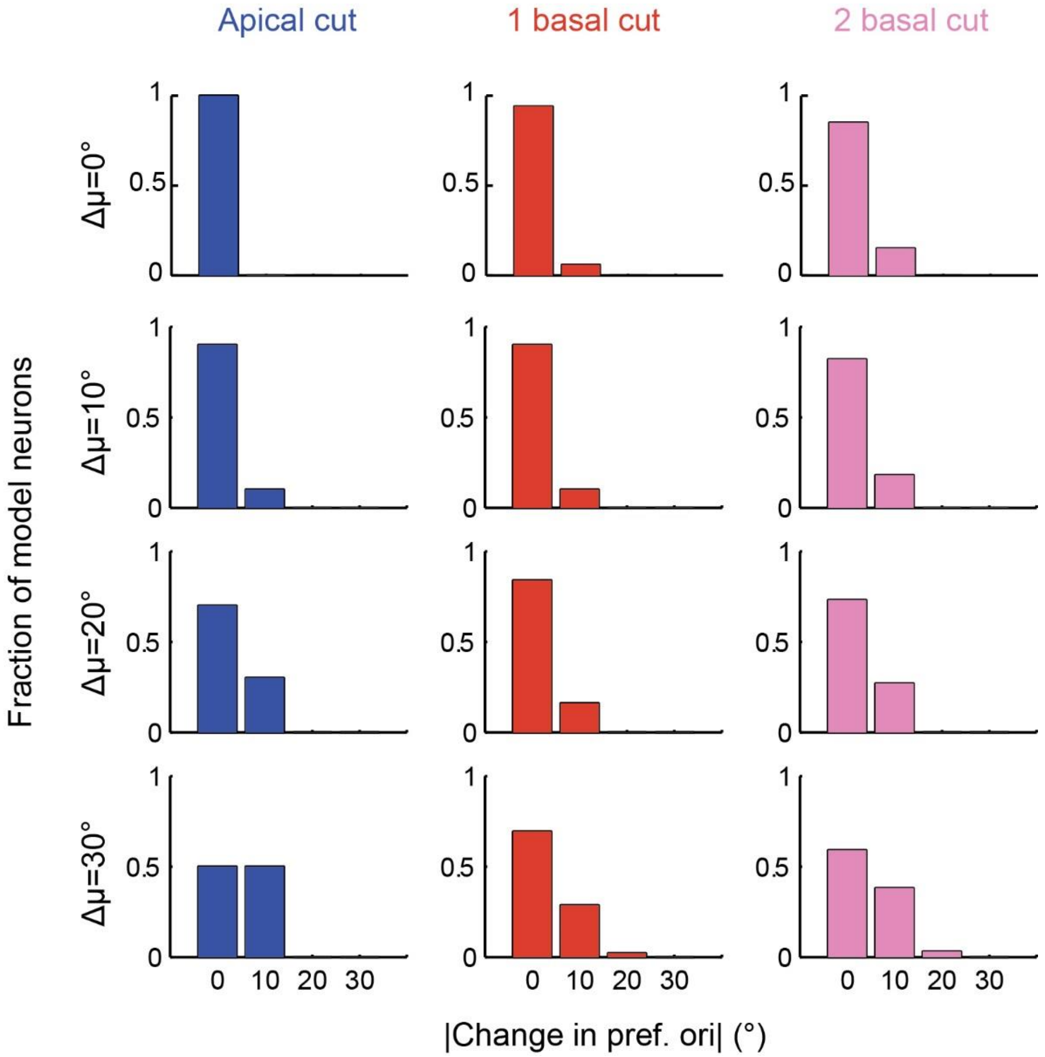

Supplementary Figure 13. Biophysical model with broadly tuned basal dendrites (drift model). Figure in the format of Figure 5b but for σ_{basal}=30°. Again, σ_{apical} is fixed at 30°. Gradually increasing Δμ leads to a very small shift in orientation preference with 2-basal cut that is a worse fit to experimental data than σ_{basal}=15°. For all, mean change in tuning-width is < 10° and mean change in OSI is < 0.2 (thresholds correspond to the mean+1std of the experimental data). Disparities of 40°or greater fail to generate tuning curves comparable to experiment (OSI≤0.2, tuning-width ≥80° for more than 30% of simulated neurons). For each model neuron (n=10 per Δμ), all combinations of one or two basal dendrite cuts were simulated.

Supplementary Table 2. Active conductances of model neuron

Conductance (mS cm^-2)	Soma	Apical	Basal
g_{Na}	0.505	0.303	0.303
g_{Kdr}	0.05	$1.5*10^{-3}$	$1.5*10^{-3}$
g_{Km}	$2.8*10^{-3}$	$1.27*10^{-3}$	$1.27*10^{-3}$
g_A	5.4	diameter<=0.8µm: 108<br>diameter<=0.8µm: 10.8	diameter<=0.8µm: 108<br>diameter<=0.8µm: 10.8
g_T	0.03	x<=260µm:<br>0.029*sin(0.009*x+0.88)<br>x>260µm: 0.012	$0.03+6*10^{-5}*x$
g_{HVA}	$0.05*10^{-3}$	x<=260µm:<br>$0.049*10^{-3}*\sin(0.009*x+0.88)$<br>x>260µm: $0.02*10^{-3}$	$0.05*10^{-3}+10^{-7}*x$
g_{KCa}	$2.1*10^{-3}$	$2.1*10^{-3}$	$2.1*10^{-3}$

Supplementary Table 3. Passive and active properties of the model neuron.

	Model	Cho et al., 2010
RMP, mV	-79	-78.56±1.34
IR, MΩ	123.6	125.2±8.2
τ, ms	17.3	16±0.7
AP amplitude, mV	66.1	67.8±1.8
AP threshold, mV	-41.8	-37.7±1.3
AHP, mV	17.9	13.3±0.5
P-T time, ms	38.6	55.3±2.7
AP adaptation	1.16	1.18±0.02

RMP: resting membrane potential, IR: Input Resistance measured at hyperpolarizing current (-0.04nA), AP: action potential, AHP: after hyperpolarization measured at depolarizing current (0.16nA), P-T peak-trough.

Supplementary Table 4. Synaptic parameters.

	Conductance (nS)	τ_1, ms	τ_2, ms
NMDA	1.15	2	30
AMPA	0.84	0.1	2.5
$GABA_A$	1.25	0.2	1.4

Supplementary Movie 1. Animated Z-stacks (1 micron step size) of an example neuron going through an apical dendrite ablation. The movie starts from bottom-up and then reverses to go top-down to the same point before stopping. It is best that each panel is seen separately first, before comparing. *Left Panel:* The neuron structure prior to ablation. Arrow shows the point on the apical trunk targeted for ablation. *Second Panel:* Neuron has been subjected to a single point scan targeted to the location shown by the arrow. Note that the neuron and its dendrites increased in brightness (shown here 10 minutes following the 1st point scan), likely as a result of calcium influx secondary to injury. However in this case the injury did not definitively sever the dendrite, which appears still grossly intact. Accordingly, the neuron regained its baseline fluorescence after ~30 minutes and the apical tuft dendrite remained intact (not shown here). *Third Panel:* The neuron is shown here 10 minutes following a second point scan targeted to the same location (shown by the arrow). Note that there is an interruption in fluorescence and the distal dendritic branches exhibit early beading morphology (a clear sign of successful ablation). Neurons whose dendrites were successfully ablated exhibited immediate but transient (<20 min) increase in fluorescence that included the targeted dendritic branches and the soma. The targeted dendritic branches then typically assumed a beads-on-a-string appearance prior to the disappearance of fluorescence. *Right Panel:* The neuron is shown 5 days following ablation. Note that the apical dendrite has now entirely disappeared. Note also that several other dendritic branches that were close (well within a 5μm range; see also Fig. 1c) but not connected to it, remain intact.

Supplementary Movie 2. 3D projection images of an example neuron pre- (left) and 5 days post- (right) apical dendrite ablation.

Supplementary Movie 3. Animated Z-stacks (moving coronally) of an example ablated neuron that was immunostained with anti-GFP (green) and anti-tuj1 (magenta). More detailed information is described in Supplementary Figure 3. The arrow indicates the ablation point. Note that there is no discernible disorganization of the nearby anti-tuj1 stained neuropil (the areas of absent staining appear to reflect nearby neurons that were not GFP stained). This is in agreement with a prior electron microscopy study (see Fig 2 of [1]) using a similar protocol, which demonstrated that the laser ablation lesion is highly contained within an area ~5 microns in diameter.

Supplementary Movie 4. Animated Z-stacks (1 micron step size) of an example neuron that underwent 2 basal dendrite ablation followed by an apical dendrite ablation. The movie starts from bottom-up and then reverses to go top-down to the same point before stopping. It is best that each panel is seen separately first, before comparing. *Left Panel:* the neuron prior to ablation. Note the red and purple arrow pointing to the basal dendrites to be ablated, and the cyan arrow pointing to the apical trunk origin near the soma. *Middle Panel:* Picture of the same neuron after two basal dendrites have been ablated. The red and purple arrows point to the ablation targets, showing the targeted basal dendrites have been severed from the soma. Beaded remnants of their distal branches can be seen along their prior trajectories in the vicinity of the cell. Note that the cell is highly fluorescent as the Z-stack was obtained during the ablation of the apical dendrite. *Right Panel:* The same neuron 5 days following apical dendrite ablation. The cyan arrow

indicates the apical dendrite ablation point. Note that the apical dendrite has deteriorated (the visible branches that can be seen towards the top a little below the arrow represent basal dendrites whose insertion point was close to that of the apical dendrite). Despite having lost 2 basal and the apical dendrites the cell survived and remained visually responsive and orientation-tuned (Supplementary Figure 9c).

Supplementary Methods

The morphologically detailed L2/3 V1 pyramidal neuron model (Figure 4A) was implemented in the NEURON simulation environment [4]. Model neurons are available in ModelDB (accession number: 231185). The passive properties of the model neuron were: membrane capacitance (C_m) 1 μF cm^{-2}, membrane resistance (Rm) 11000 Ω cm^2 and axial resistance (Ra) 100 Ω cm. In the basal and apical dendrites, C_m was doubled to account for dendritic spines. The resting membrane potential was set at -79mV and the input resistance (R_{IN}) at 124 MΩ [5–7]. Membrane time constant was 17 ms [6].

The active properties of the model were adapted from [7,8]. The model included conductances for fast voltage-dependent sodium channels (g_{Na}), delayed rectifier potassium channels (g_{kdr}), slow voltage-dependent potassium channels (g_M), A-type potassium channels (g_A), calcium-activated potassium channels (g_{KCa}) and high and low voltage-activated calcium channels (g_{HVA} and g_T respectively). In all compartments a calcium buffering mechanism was included (τ=50ms). At the apical dendrites the conductances of the calcium channels increased for distances <=80μm and then decreased and reached minimal levels for distances above 260μm. At the basal dendrites, calcium channel conductances increased linearly [9]. Compared to the conductance of the thin dendrites (diameter<=0.8), the conductance of the A-type potassium channel was 5% at the soma and 10% at the thick dendrites [10]. Supplementary Table 2 shows the ionic conductances used in each compartment. Supplementary Table 3 lists the electrophysiological characteristics of the model neuron in comparison to experimental data of [6]. Supplementary Figure 10a illustrates the electrophysiological profile of the model neuron in comparison to an experimental trace adapted from [2] in response to the same current step pulse (0.16 nA). The model is in great agreement with all of the abovementioned experimental data.

Synaptic mechanisms included AMPA, NMDA and $GABA_A$ synapses. Kinetics of miniature AMPA current (activation of 1 synapse) were fitted to experimental data of [11]. In particular, the model neuron was voltage clamped at -70mV and consecutively 1 AMPA synapse was activated at each dendritic branch. The average response of the model (red trace) as well as the experimental average response (in the presence of APV to isolate the AMPA current) is shown in the right panel of Supplementray Figure10a. The kinetics of the NMDA current were also fitted to the experimental data of [11] (Supplementary Figure 10a, green trace) in the absence of Mg^{++} blockade, to simulate the experimental absence of Mg^{++} (only for validation purposes). The peak amplitude of the NMDA current was set so that the ratio of the iNMDA (measured at +50mV) to iAMPA current (measured at -50mV) was 1.2 [11]. This resulted in gNMDA=1.37*gAMPA. Miniature EPSC amplitude (iAMPA and iNMDA) was set at 20 pA [11]. The unitary IPSP (activation of 15 inhibitory synapses) was 0.8 mV measured at resting membrane potential -65mV and the duration at half amplitude was set to 15ms [12]. Properties of synaptic currents used are shown in Supplementary Table 4.

To estimate the total number of excitatory synapses, we assumed synaptic density of 2 excitatory synapses (spines)μm^-1 [13,14]. Excitatory synapses consisted of both AMPA and NMDA conductances. The total dendritic length of the model neuron is 3298μm and thus the total number of excitatory synapses was 6596, that is within the range of the experimentally reported total number of excitatory synapses in layer 2/3

visual cortex [13]. 60% of these were randomly distributed to the basal dendrites and 40% to the apical dendrites [13]. Distribution of synapses to individual dendritic compartments was adjusted so that each dendrite had the same synaptic density, that is, the number of synapses each dendrite received was proportional to its length. 15% of the total number of synapses were inhibitory [13,15]. 7% of inhibitory synapses were located at the soma, 60% at the basal dendrites and 33% at the apical dendrites [16]. 25% of the total number of excitatory synapses were stimulus-driven [3]. The rest 75% of excitatory synapses, as well as the inhibitory synapses [17], were independently driven by Poisson spike trains with mean frequency 0.11Hz (Supplementray Figure 10b). This resulted in spontaneous spiking activity at frequency 0.28±0.37 Hz that is within the experimental range from in vivo recordings in mice [18].

Each stimulus-driven synapse was tuned (i.e. had a preferred orientation, $pref_{syn}$) in one of the thirty-six different angles (0°-350° with step 10°). Tuning was not direction selective i.e. a synapse with tuning at 0° was also tuned at 180°. The orientation-preference distribution of the stimulus-synapses followed the sum of two Gaussians centered on the mean orientation preference (orientation preference with maximum probability) of the dendritic tree (μ_{tree}). We varied the standard deviations (σ_{basal}, σ_{apical}) of the distributions from 15° to 30°, 45° and 60° (Supplementary Figure 10c). For σ=30°, distributions resemble the ones reported in [3]. In addition, we ranged the difference (Δ) of μ_{basal} and μ_{apical} from 0° to 90°, with step 10° (by varying the μ_{basal} and arbitrary keeping constant the μ_{apical} at 0°, Supplementary Figure 10d). In the drift model, synapses of each basal dendrite (and not the whole dendritic tree) were assigned a preference according to the respective μ_b, σ_b (Fig. 5A). The μ_b of each dendrite was chosen from a predefined range (Δ_μ).

60% of orientation-tuned synapses were randomly dispersed to basal dendrites and the rest 40% to the apical dendrites [13,19] (Supplementary Figure 10d), so that all dendrites have the same density of stimulus synapses. Stimulus (a bar at a specific direction) was 'presented' after an initial period of 500ms and lasted for 2 secs as in the experimental setup (Fig. 4b). During the 'presentation' of the moving bar (0°-180°, with step 10°), stimulus-driven synapses were activated by Poisson spike trains (Supplementary Figure 10e). Each stimulus-driven synapse received a spike train with mean 0.3 Hz*activation factor, where the activation factor depended on the Δ(stimulus, $pref_{syn}$) (Supplementary Figure 10e). The neuron responded under the control conditions (see below) for the preferred orientation with 1.65 ±0.94 Hz [18]. Control condition was the one corresponding to $\sigma_{apical}=\sigma_{basal}=30°$, $\mu_{apical}=\mu_{basal}=0°$, $\Delta\mu=0°$. Under this condition, the OSI was 0.78 and the tuning width 43° (Fig. 4c), replicating the experimental data for orientation tuning in L2/3 neurons of the visual cortex.

Simulation of the apical tree and basal dendrite ablation was performed by removing the respective compartments. We simulated basal dendrite cutting by removing each one of the five primary basal dendrites for one dendrite cutting or all combinations of two primary basal dendrites for two dendrites cutting. The model neuron's firing rate increased following apical dendrite ablation due to a 2.5-fold increase in input resistance (as seen in [20]), while basal dendrite ablation slightly decreased firing rate due to excitatory input loss. In the model, synaptic scaling (reduction) of excitatory transmission was implemented (as observed in prior studies of homeostatic plasticity [21]) to normalize the neuron's firing rate without affecting the tuning curve shape. Specifically, pre-/post-

ablation excitatory synaptic weights (gAMP and gNMDA) were adjusted to result in the same levels of spiking activity at the preferred and the orthogonal orientation under control condition, as indicated by the calcium data (Supplementary Figure 6).
For each condition, we simulated 10 model neurons (different in respect to the $pref_{syn}$ of each synapse, as well as in their location along the trees) and for each neuron we averaged the resulting spiking activity over 10 repetitions. For each condition, preferred orientation at the cell body was defined as the orientation (0° to 170° with step 10°) to which the neuron displayed the highest mean firing frequency (Supplementary Figure 10e). Since we did not model direction selectivity, each preferred orientation was also represented by its symmetric angle (e.g. 0° and 180°). Accordingly, the orientation tuning-width was the width at half amplitude of the fitted curve. Selectivity of orientation tuning for each condition was assessed using the OSI metric, defined as $OSI = \frac{R_{pref}-R_{ortho}}{R_{pref}+R_{ortho}}$. Simulated neurons that lacked realistic tuning curves (OSI ≤ 0.2, tuning width ≥ 80°) were excluded from the analysis. We further did not consider orientation tuning for combinations of simulation parameters in which more than 30% of the simulated neurons were categorized as not tuned.
In Figure 4F, mode change was plotted for orientation preference and average change was used for plotting changes in tuning-width and OSI.

In the drift model, $\Delta\mu$ of 50° or greater failed to generate tuning curves comparable to experiment (>30% of simulated cells), so the analysis was limited to $\Delta\mu$=20-40°.

To assess if the change in input structure across basal dendrites following two basal dendrite ablation in fact relates to the change in orientation preference of the model neuron, we calculated $\widetilde{\Delta\mu}_{ablated}$, the difference between length normalized orientation preference in input structure across basal dendrites before and after two basal dendrite ablation. The $\widetilde{\Delta\mu}_{ablated}$ was calculated for each combination of ablated dendrites as follows:

For each primary basal dendrite,

$$\vec{\mathbf{r}}_{\mathbf{i}} = \frac{L_i}{L_{max}} e^{j\theta_i} \quad (1)$$

, where L_i is the length of the i_{th} dendrite normalized to the length of the longest dendrite (L_{max}) and θ_i the mean of orientation preference of the distribution of synapses of the respective basal dendrite (in radians). The population vector, using Euler's formula, is:

$$\vec{\mathbf{r}} = \sum_i^n \frac{L_i}{L_{max}} \cos\theta_i + j \sum_i^n \frac{L_i}{L_{max}} \sin\theta_i \quad (2)$$

, where n is the number of primary basal dendrites. The angle of the population vector is:

$$\varphi = \tan^{-1} \frac{\sum_i^n \frac{L_i}{L_{max}} \sin\theta_i}{\sum_i^n \frac{L_i}{L_{max}} \cos\theta_i} \quad (3)$$

Finally, we defined $\widetilde{\Delta\mu}_{ablated} = |\varphi_{pre} - \varphi_{post}|$
,where φ_{pre} is the population vector of the input structure before the ablation of the primary basal dendrites and φ_{post} after (in degree).

Simulations were performed in the Computational Biology Lab High Performance Computational (HPC) cluster consisting of 312 High Performance CPU cores and 1.150 Gigabytes of RAM. Analysis was performed in Python 2.7.13 via Anaconda 4.4.0. The model neuron code is available in ModelDB (accession number 231185).

References

1. Canty, A. J. *et al.* In-vivo single neuron axotomy triggers axon regeneration to restore synaptic density in specific cortical circuits. *Nat. Commun.* **4**, (2013).
2. Rhie, D. *et al.* Electrophysiological and Morphological Classification of Inhibitory Interneurons in Layer II / III of the Rat Visual Cortex. *Korean J. Physiol. Pharmacol.* **7**, 317–323 (2003).
3. Chen, T.-W. *et al.* Ultrasensitive fluorescent proteins for imaging neuronal activity. *Nature* **499**, 295–300 (2013).
4. Hines, M. L. L. & Carnevale, N. T. Neuron: A Tool for Neuroscientists. *Neurosci.* **7**, 123–135 (2001).
5. Cho, K.-H. *et al.* Differential cholinergic modulation of Ca2+ transients evoked by backpropagating action potentials in apical and basal dendrites of cortical pyramidal neurons. *J. Neurophysiol.* **99**, 2833–43 (2008).
6. Cho, K.-H. *et al.* Subtype-specific dendritic Ca(2+) dynamics of inhibitory interneurons in the rat visual cortex. *J. Neurophysiol.* **104**, 840–853 (2010).
7. Smith, S. L., Smith, I. T., Branco, T., Häusser, M. & Hausser, M. Dendritic spikes enhance stimulus selectivity in cortical neurons in vivo. *Nature* **503**, 115–120 (2013).
8. Branco, T., Clark, B. A. & Hausser, M. Dendritic Discrimination of Temporal Input Sequences in Cortical Neurons. *Science (80-.).* **329**, 1671–1675 (2010).
9. Cho, K. *et al.* Spatial profile of back-propagating action potential-evoked Ca2+ transients in basal dendrites. *Neuroreport* **17**, 131–134 (2006).
10. Burkhalter, A., Gonchar, Y., Mellor, R. L. & Nerbonne, J. M. Differential expression of I(A) channel subunits Kv4.2 and Kv4.3 in mouse visual cortical neurons and synapses. *J. Neurosci.* **26**, 12274–12282 (2006).
11. Myme, C. I. O., Sugino, K., Turrigiano, G. G. & Nelson, S. B. The NMDA-to-AMPA ratio at synapses onto layer 2/3 pyramidal neurons is conserved across prefrontal and visual cortices. *J. Neurophysiol.* **90**, 771–9 (2003).
12. Tamás, G., Buhl, E. H. & Somogyi, P. Fast IPSPs elicited via multiple synaptic release sites by different types of GABAergic neurone in the cat visual cortex. *J. Physiol.* **500 (Pt 3**, 715–738 (1997).
13. DeFelipe, J. & Farinas, I. The pyramidal neuron of the cerebral cortex: morphological and chemical characteristics of the synaptic inputs. *Prog Neurobiol* **39**, 563–607 (1992).
14. Schüz, A. & Palm, G. Density of neurons and synapses in the cerebral cortex of the mouse. *J. Comp. Neurol.* **286**, 442–55 (1989).
15. Binzegger, T., Douglas, R. J. & Martin, K. A. A quantitative map of the circuit of cat primary visual cortex. *J Neurosci* **24**, 8441–8453 (2004).
16. DeFelipe, J. *et al.* Neocortical circuits: evolutionary aspects and specificity versus non-specificity of synaptic connections. Remarks, main conclusions and general comments and discussion. *J. Neurocytol.* **31**, 387–416 (2003).
17. Gentet, L. J., Avermann, M., Matyas, F., Staiger, J. F. & Petersen, C. C. H. Membrane potential dynamics of GABAergic neurons in the barrel cortex of behaving mice. *Neuron* **65**, 422–35 (2010).

18. Haider, B., Häusser, M. & Carandini, M. Inhibition dominates sensory responses in the awake cortex. *Nature* **493**, 97–100 (2013).
19. Jia, H., Rochefort, N. L., Chen, X. & Konnerth, A. Dendritic organization of sensory input to cortical neurons in vivo. *Nature* **464**, 1307–1312 (2010).
20. Bekkers, J. M. & Hausser, M. Targeted dendrotomy reveals active and passive contributions of the dendritic tree to synaptic integration and neuronal output. *Proc Natl Acad Sci U S A* **104**, 11447–11452 (2007).
21. Turrigiano, G. G. The Self-Tuning Neuron: Synaptic Scaling of Excitatory Synapses. *Cell* **135**, 422–435 (2008).
