# Supplementary material for: Contribution of apical and basal dendrites to orientation encoding in mouse V1 L2/3 pyramidal neurons
Source: Nat Commun. 2019 Nov 26;10:5372. doi: 10.1038/s41467-019-13029-0 (PMC6879601; doi:10.1038/s41467-019-13029-0)
Supplement: Supplementary file 3 — Reporting Summary [file 41467_2019_13029_MOESM3_ESM.pdf]

nature research

Corresponding author(s): NCOMMS-18-24521B

Last updated by author(s): Sep 6, 2019

Reporting Summary

Nature Research wishes to improve the reproducibility of the work that we publish. This form provides structure for consistency and transparency in reporting. For further information on Nature Research policies, see Authors & Referees and the Editorial Policy Checklist .

Statistics

For all statistical analyses, confirm that the following items are present in the figure legend, table legend, main text, or Methods section.

n/a	Confirmed	
☐	☒	The exact sample size (n) for each experimental group/condition, given as a discrete number and unit of measurement
☐	☒	A statement on whether measurements were taken from distinct samples or whether the same sample was measured repeatedly
☐	☒	The statistical test(s) used AND whether they are one- or two-sided<br>*Only common tests should be described solely by name; describe more complex techniques in the Methods section.*
☐	☒	A description of all covariates tested
☐	☒	A description of any assumptions or corrections, such as tests of normality and adjustment for multiple comparisons
☐	☒	A full description of the statistical parameters including central tendency (e.g. means) or other basic estimates (e.g. regression coefficient) AND variation (e.g. standard deviation) or associated estimates of uncertainty (e.g. confidence intervals)
☐	☒	For null hypothesis testing, the test statistic (e.g. F, t, r) with confidence intervals, effect sizes, degrees of freedom and P value noted<br>*Give P values as exact values whenever suitable.*
☒	☐	For Bayesian analysis, information on the choice of priors and Markov chain Monte Carlo settings
☒	☐	For hierarchical and complex designs, identification of the appropriate level for tests and full reporting of outcomes
☒	☐	Estimates of effect sizes (e.g. Cohen's d, Pearson's r), indicating how they were calculated

Our web collection on statistics for biologists contains articles on many of the points above.

Software and code

Policy information about availability of computer code

Data collection	Prairie View Software was used to collect two photon images and other experiment parameters such as time stamps of onset/offset of the visual stimuli.
Data analysis	We used Matlab R2017a to analyze the data.

For manuscripts utilizing custom algorithms or software that are central to the research but not yet described in published literature, software must be made available to editors/reviewers. We strongly encourage code deposition in a community repository (e.g. GitHub). See the Nature Research guidelines for submitting code & software for further information.

Data

Policy information about availability of data

All manuscripts must include a data availability statement. This statement should provide the following information, where applicable:

- Accession codes, unique identifiers, or web links for publicly available datasets
- A list of figures that have associated raw data
- A description of any restrictions on data availability

Raw datasets associated with Figure 2 and 3 will be available from the corresponding authors on request. The biophysical model used during the current study is available in the ModelDB repository with the accession number 231185.

Field-specific reporting

Please select the one below that is the best fit for your research. If you are not sure, read the appropriate sections before making your selection.

Life sciences study design

All studies must disclose on these points even when the disclosure is negative.

Sample size	Power analysis was used to decide number of neurons needed to detect the statistical difference in orientation tuning post ablation.
Data exclusions	Extremely poorly tuned neurons with peak calcium response (GCaMP6s signal) less than 2XSEM of the calcium responses to the null orientation were excluded from the analysis.
Replication	We confirmed our major results (both apical and basal dendrite ablation) with two different microscope setups (one in BCM, Baylor College of Medicine and another in BWH, Brigham and Women's Hospital) using two independent groups of mice (both purchased from Jackson Lab initially then bred for experiments accordingly at BCM and BWH). There was no differences between data collected from BCM and BWH.
Randomization	Neurons with strong tuning (OSI>0.3) and clear apical trunk (>20um) located deeper than 150um were chosen as ablation candidates. Neurons imaged together with the ablation candidate served as control neurons. Control neurons were pyramidal neurons (labeled with CamKII-Cre) located on the same depth with the ablation candidate. Control and experiment groups had similar OSIs and other tuning properties.
Blinding	Orientation tuning curve analysis was performed blind to ablation identity.

Reporting for specific materials, systems and methods

We require information from authors about some types of materials, experimental systems and methods used in many studies. Here, indicate whether each material, system or method listed is relevant to your study. If you are not sure if a list item applies to your research, read the appropriate section before selecting a response.

Materials & experimental systems

n/a	Involved in the study	
☐	☒	Antibodies
☒	☐	Eukaryotic cell lines
☒	☐	Palaeontology
☐	☒	Animals and other organisms
☒	☐	Human research participants
☒	☐	Clinical data

Methods

n/a	Involved in the study	
☒	☐	ChIP-seq
☒	☐	Flow cytometry
☒	☐	MRI-based neuroimaging

Antibodies

Antibodies used	Chicken anti GFP polyclonal (ABCAM: ab13970, primary antibody);<br>Mouse anti BetaIII Tubulin Monoclonal (Santa Cruz: SC-58888, primary antibody);<br>Goat anti chicken IgY (H+L) Secondary Antibody Alexa Fluor 488 (CAT: A-11039);<br>Goat anti mouse IgG (H+L) Cross-Absorbed Secondary Antibody, Alexa Fluor 594 (CAT: A-11005)
Validation	anti GFP: https://www.abcam.com/gfp-antibody-ab13970.html ;<br>anti BetaIII Tubulin: https://www.citeab.com/antibodies/835128-sc-58888-3-tubulin-antibody-tuj-1 ;<br>Goat anti chicken (A-11039):<br>https://www.thermofisher.com/antibody/product/Goat-anti-Chicken-IgY-H-L-Secondary-Antibody-Polyclonal/A-11039 ;<br>Goat anti mouse (A-11005):<br>https://www.thermofisher.com/antibody/product/Goat-anti-Mouse-IgG-H-L-Cross-Adsorbed-Secondary-Antibody-Polyclonal/A-11005

Animals and other organisms

Policy information about studies involving animals; ARRIVE guidelines recommended for reporting animal research

Laboratory animals	Male and female wild-type (C57BL/6) mice of 8-16wk old were used
Wild animals	The study did not involve wild animals
Field-collected samples	The study did not collect samples collected from the field
Ethics oversight	All experimental protocols were approved by The Baylor College of Medicine (BCM) Institutional Review Board and Brigham and Women's Hospital (BWH) Institution Animal Care and Use Committee.

Note that full information on the approval of the study protocol must also be provided in the manuscript.
