# Supplementary material for: Contribution of apical and basal dendrites to orientation encoding in mouse V1 L2/3 pyramidal neurons
Source: Nat Commun. 2019 Nov 26;10:5372. doi: 10.1038/s41467-019-13029-0 (PMC6879601; doi:10.1038/s41467-019-13029-0)
Supplement: Supplementary file 4 — Description of Additional Supplementary Files [file 41467_2019_13029_MOESM4_ESM.pdf]

Description of Additional Supplementary Files

File Name: Supplementary Movie 1
Description: Animated Z-stacks (1 micron step size) of an example neuron going through an apical dendrite ablation. The movie starts from bottom-up and then reverses to go top-down to the same point before stopping. It is best that each panel is seen separately first, before comparing. Left Panel: The neuron structure prior to ablation. Arrow shows the point on the apical trunk targeted for ablation. Second Panel: Neuron has been subjected to a single point scan targeted to the location shown by the arrow. Note that the neuron and its dendrites increased in brightness (shown here 10 minutes following the 1st point scan), likely as a result of calcium influx secondary to injury. However in this case the injury did not definitively sever the dendrite, which appears still grossly intact. Accordingly, the neuron regained its baseline fluorescence after ~30 minutes and the apical tuft dendrite remained intact (not shown here). Third Panel: The neuron is shown here 10 minutes following a second point scan targeted to the same location (shown by the arrow). Note that there is an interruption in fluorescence and the distal dendritic branches exhibit early beading morphology (a clear sign of successful ablation). Neurons whose dendrites were successfully ablated exhibited immediate but transient (<20 min) increase in fluorescence that included the targeted dendritic branches and the soma. The targeted dendritic branches then typically assumed a beads-on-a-string appearance prior to the disappearance of fluorescence. Right Panel: The neuron is shown 5 days following ablation. Note that the apical dendrite has now entirely disappeared. Note also that several other dendritic branches that were close (well within a 5μm range; see also Fig. 1c) but not connected to it, remain intact.

File Name: Supplementary Movie 2
Description: 3D projection images of an example neuron pre- (left) and 5 days post- (right) apical dendrite ablation.

File Name: Supplementary Movie 3
Description: Animated Z-stacks (moving coronally) of an example ablated neuron that was immunostained with anti-GFP (green) and anti-tuj1 (magenta). More detailed information is described in Supplementary Figure 3. The arrow indicates the ablation point. Note that there is no discernible disorganization of the nearby anti-tuj1 stained neuropil (the areas of absent staining appear to reflect nearby neurons that were not GFP stained). This is in agreement with a prior electron microscopy study (see Fig 2 of 1) using a similar protocol, which demonstrated that the laser ablation lesion is highly contained within an area ~5 microns in diameter.

File Name: Supplementary Movie 4
Description: Animated Z-stacks (1 micron step size) of an example neuron that underwent 2 basal dendrite ablation followed by an apical dendrite ablation. The movie starts from bottom-up and then reverses to go top-down to the same point before stopping. It is best that each panel is seen separately first, before comparing. Left Panel: the neuron prior to ablation. Note the red and purple arrow pointing to the basal dendrites to be ablated, and the cyan arrow pointing to the apical trunk origin near the soma. Middle Panel: Picture of the same neuron after two basal dendrites have been ablated. The red and purple arrows point to the ablation targets, showing the targeted basal dendrites have been severed from the soma. Beaded remnants of their distal branches can be seen along their prior trajectories in the vicinity of the cell. Note that the cell is highly fluorescent as the Z-stack was obtained during the ablation of the apical dendrite. Right Panel: The same neuron 5 days following apical dendrite ablation. The cyan arrow indicates the apical dendrite ablation point. Note that the apical dendrite has deteriorated (the visible branches that can be seen towards the top a little below the arrow represent basal dendrites whose insertion point was close to that of the apical

dendrite). Despite having lost 2 basal and the apical dendrites the cell survived and remained visually responsive and orientation-tuned (Supplementary Figure 9c).
